# Daily activity patterns and body temperature of the Oriental migratory locust, *Locusta migratoria manilensis* (Meyen), in natural habitat

**DOI:** 10.3389/fphys.2023.1110998

**Published:** 2023-02-03

**Authors:** Hongmei Li, Jingquan Zhu, Yumeng Cheng, Fuyan Zhuo, Yinmin Liu, Jingfeng Huang, Bryony Taylor, Belinda Luke, Meizhi Wang, Pablo González-Moreno

**Affiliations:** ^1^ MARA-CABI Joint Laboratory for Bio-safety, Institute of Plant Protection, Chinese Academy of Agricultural Science, Beijing, China; ^2^ CABI East and Southeast Asia, Beijing, China; ^3^ National Agro-Tech Extension and Service Center, Beijing, China; ^4^ College of Environment and Resources Science, Zhejiang University, Hangzhou, China; ^5^ CABI, Egham, United Kingdom; ^6^ Department of Forest Engineering, ERSAF RNM-360, University of Córdoba, Córdoba, Spain

**Keywords:** *Locusta migratoria manilensis*, locusts, body temperature, entomopathogenic fungus, body length, position, development

## Abstract

Current pest management techniques would benefit from understanding the behavioural rhythms of the target pest and its body temperature, a critical aspect not well studied and potentially limiting the effectiveness of biopesticides under natural conditions. This study aims 1) to understand under natural conditions the behavioural patterns of different stages of hoppers and adults of *Locusta migratoria manilensis* and 2) to identify the environmental factors modulating their body temperature through field observation. We carried out an intensive field sampling in two of the main locust breeding regions in China, recording the body temperature (day and night), morphological traits (stage, sex and size) and microhabitat of 953 individuals. The results revealed that locusts preferred the ground as their main activity subhabitat, particularly for hoppers. Adults tended to move upper in the reed canopy at two peaks (10-11 h and 14-15 h). Locusts body temperature during daytime increased with development stage and size, while the opposite pattern occurred during night time. Entompathogenic fungi are more effective if the body temperature of the target pest is in a proper range without too high or too low. Application of biopesticides should focus on younger locusts spraying in the morning or at dusk as the locusts have lower body temperatures.

## 1 Introduction

Locust plagues cause problems globally in almost all ecosystems except the forest and tundra belts in the north and the equatorial forests. Heavy locust infestations have been often reported in various parts of the world causing enormous damage to agriculture and the environment but without a constant pattern. For instance, locust swarms were reported causing damage to agriculture in New South Wales, Australia in 2015 (Jurd, 2015). Due to the high potential hazard, all countries in West Africa continue maintaining a high standard of reporting that is the basis for the global Desert Locust early warning system. Desert locust, *Schistocerca gregaria* (Forskål), has been causing serious problems in areas about 29 million km^2^ from the Atlantic Ocean in the west to India and Pakistan in the east, and comprising the entire area or parts of 64 countries (Harb, 2009). In China, locust’ populations have been stable and no major outbreaks have occurred in recent years. Nevertheless, there are still localised spots of high locust density in marshlands of Jilin, Shanxi and Shandong Provinces (Yang et al., 2018). Specifically, Oriental migratory locust, *Locusta migratoria manilensis*, is one of the key locust species in China occasionally causing destructive outbreaks both in frequency and scale (Xia and Huang, 2002).

Chemical pesticides are the main intervention used against locusts and grasshoppers. Extensive applications of insecticides, particularly organophosphates, have inevitably resulted in the development of resistance in natural populations of the locusts and damage to biodiversity (Zhang, 2011). Preventive management and usage of biopesticides are garnering increasing attention from the government as partial alternatives for chemical pesticides (Ndolo et al., 2019). Many studies of locust biocontrol made great progress, and alternative microbial biopesticides such as those based on entomopathogenic fungus (EPF) greatly reduce the risk of resistance and pollution. *Metarhizium acridum* is an EPF with a narrow host range comprising locusts and grasshoppers, and proofed that *M. acridum* strains had a strong virulence to *L. m. manilensis* (Cao et al., 2016). Strains of *M. acridum* have been developed as biopesticide against locust and grasshoppers worldwide in Africa, Australia, China, Brazil and Mexico (Bateman, 1997; Driver et al., 2000; Peng and Xia, 2011; Maute et al., 2015), including Isolate IMI 330189 marketed as Green Muscle^®^, IMI189.

Conidia of *M. acridum* require suitable environmental conditions in terms of humidity and temperature to survive and germinate to infect the insect host. The internal temperature of the locust strongly influences the rate of fungal development and the ultimate time to death of the insect (Klass et al., 2007). Studies investigating the relationship between temperature and rate of growth of fungal pathogens have shown that there is a non-linear relation between EPF development and temperature with the lower development threshold for *M. acridum* reported around 10–12°C and the upper around 35–37°C (Smits et al., 2003; Klass et al., 2007; Yao et al., 2007). Above the upper development threshold, growth of the fungus is halted until temperatures fall below the threshold. The optimal growth rate has been shown to be around 28°C. There are many examples of temperature-dependent mortality rates for EPFs used in the biocontrol of insect pests (Doberski, 1981; Carruthers et al., 1985). These findings suggest that environmental conditions are an important factor to the success of microbial control and brings into question the assumption of constant time to death irrespectively of the context. Furthermore, many grasshoppers and locusts can behaviourally regulate their body temperature well above ambient over large parts of the day during sunny conditions (Chappell and Whitam, 1990). This behaviour has been shown to slow disease incubation and confer significant survival advantage (Carruthers et al., 1992; Inglis et al., 1996; Blanford et al., 1998; Blanford and Thomas, 1999a; b). In field trials, mortality does not usually occur earlier than 6 days after spraying and may take longer (Bateman, 1997). Speed of kill is influenced strongly by dose and environmental conditions and their insect hosts, e.g., internal body temperatures of the host insect. Therefore, to encourage the wider use of biopesticides, it is vital to understand how the locust body temperature fluctuates under different habitats and locust characteristics, so that biopesticide spraying can take place when conditions are most favourable to the pathogen.

The body temperature of locusts and grasshoppers is influenced by microclimate, adjacent vegetation, terrain and weather including solar radiation, wind and clouds (Willmer, 1982; Coxwell and Bock, 1995). Moreover, their posture, body colour can also lead to different body temperatures under the same environmental conditions (Stevenson, 1985; Jong et al., 1996; Miller and Denny, 2011). Most insects, such as locusts and grasshoppers, have limited capacity to internally regulate their body temperature and mainly rely on the surrounding environment (Angilletta, 2009). Microhabitat selection is predicted to have the greatest effect on an ectotherm’s temperature (Stevenson, 1985). Temperatures of plant canopy exert the principal effect on body temperature of *L. m. manilensis*, especially the ground temperature (Liu et al., 2018).

Although previous laboratory work (Blandford and Thomas, 1999a; Arthurs and Thomas 2000; Yao et al., 2007; Yue et al., 2010; Liu et al., 2019) has expanded our knowledge and provided an invaluable information about the relation between temperature and the effectiveness of EPFs on insect control, there is a need for complementary studies involving more realistic environmental conditions. Behavioural and physiological rhythms in a natural setting remain relatively unexplored for many species, which will provide valuable insights into the precise pest control including time and target location of field treatments (Miyata, 2011). Here, we use *L. m. manilensis* in the main breeding regions of China as a case study to understand daily activity rhythms and behaviour of *L. m. manilensis* under real field conditions *via* the detection of body temperature and locust traits. We conducted extensive field surveys recording locust body characteristics and habitat in two key locust breeding zones, which were lake-reservoir type/wetland and coastal type of locusts breeding habitats on the east coast of China. Specifically, we aimed to answer the following questions: 1) do locust prefer different reed habitat across development stage and location? 2) how body and ambient temperature fluctuates during the day across locations? 3) does body temperature differ across study area, development stage, reed habitat, sex, and body length? Upon the strength of these relations, we could generalize body temperature patterns of locusts across different locations under similar habitat condition. We further discuss how these results may help to tailor the biopesticide product treatment based on environment characteristics, in order to improve the efficacy of the control measures.

## 2 Materials and methods

### 2.1 Study zones

**FIGURE 1:**
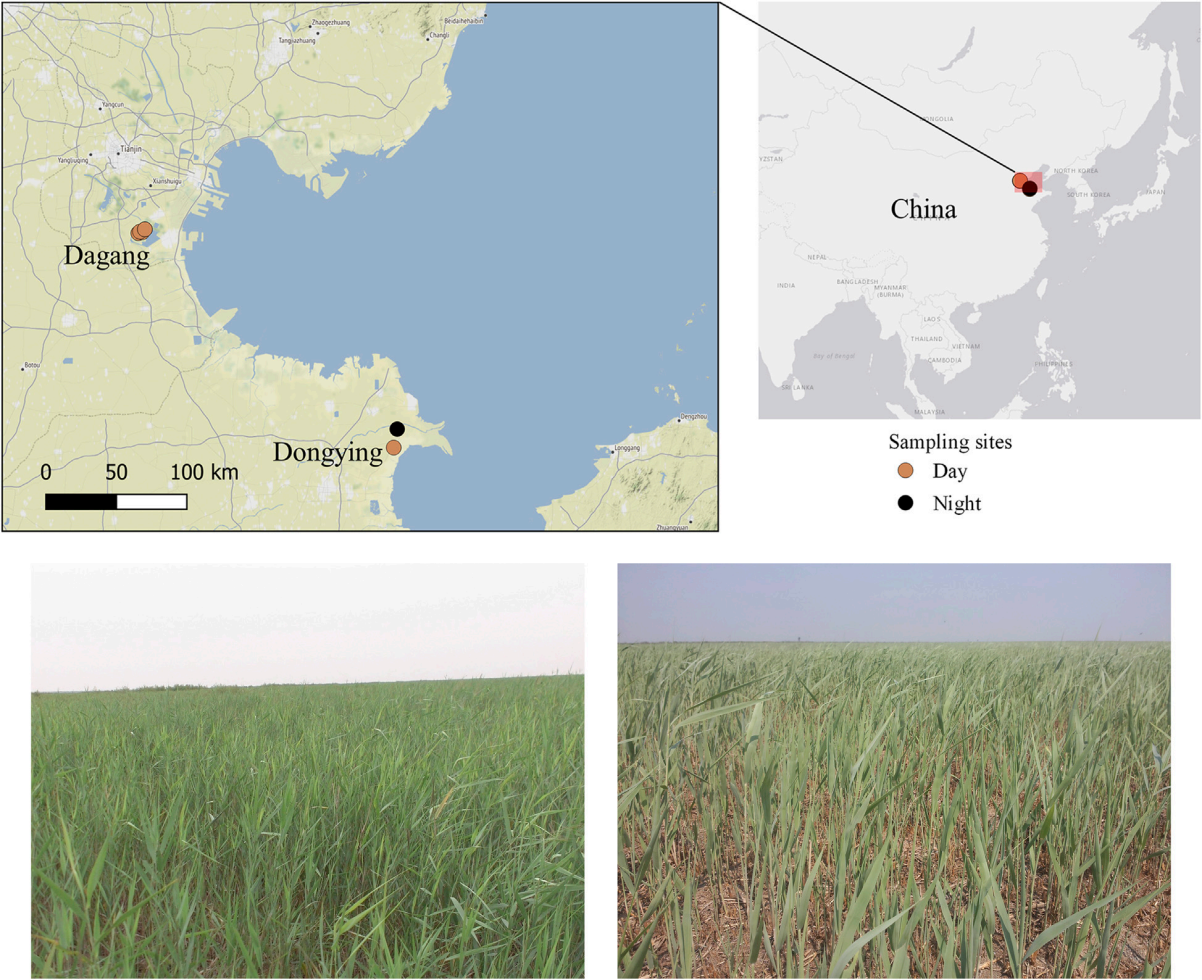
Map of study areas (Dagang and Dongying) on the East coast of China.

Two experimental zones were selected, which represent lake-reservoir type/wetland and coastal type of locusts breeding habitats on the east coast of China (Figure 1): Dagang (DAG) and Dongying (DON). Dagang study area was located in the North Dagang Reservoir area at the southeast of Tianjin City. There is an area of approximately 45,000 ha suitable for locusts (Fan et al., 2010). Dongying study area corresponds to the Yellow River Delta area (Kenli district, Shandong Province, China). This area is a long-known breeding area for locusts in China, and the Kenli district is one of the key locust regions, where there are approximately 148,000 ha suitable for locust occurrence (Hu, 2018). The main soil types in both regions are tidal soil, saline soil and coastal saline. Local farmers will choose slight alkaline land to plant agricultural crops, such as maize, rice, peanut, or establish ponds to culture lotus and feed fish and shrimps in the wetland. These two zones belong to non-agricultural land, and are full of bulrush and gramineous vegetations, and the dominant plants in the experimental areas are *Phragmites australis*, mainly associated with *Suaeda glauca*, *Alternanthera sessilis*, *Cynodon dactylon* and *Aster subulatus*. *L. m. manilensis* is the main locust species in both zones producing up to two generations a year. We selected similar sites in terms of vegetation for both zones: four in Dagang and two in Dongying (Figure 1).

### 2.2 Body temperature and insect traits sampling

Data were collected in Dagang during summer 2016 and in Dongying in summer 2017 (i.e., June, July and August). We conducted two different survey methods, during the day (6:00–18:59) and night time (19:00–5:59). During day time we collected the insects in open field conditions either hand caught or trapped in a small sweep net. Each site was visited weekly until at least five insects were recorded per hour. During the night, due to safety reasons, we were not able to sample in open field conditions. Thus, about 100 locust nymphs were collected in advance during the day and placed in a cage (2 m× 2 m× 2 m) with natural reedbed vegetation at a field station (Figure 1). For practical reasons, we could only carry out this night experiment in Dongying area. A minimum of 10 insects per hour were hand caught randomly chosen from the cage. A total of 645 and 308 locusts were collected during day and night sampling respectively.

In both day and night surveys, internal body temperatures were recorded using a 0.125 mm diameter copper thermocouple connected to a hand-held, fast response, digital thermometer (type 0.1°C resolution, 5 s response time, −0.1 mm diameter; Eutech Instruments). For each insect caught, we made a small hole in its thorax with the tip of a 0.22-mm diameter hypodermic needle (Blandford et al., 1998). A thermocouple was then inserted to a depth of 2 mm and a reading taken at the point the temperature stabilized. Insects were discarded and recordings were not taken when capture and/or length of time for the recording to be taken was felt to have affected the real body temperature (10–12 s after capture). Generally, insects were processed within 5–7 s of capture. Besides internal body temperature, we also recorded the time (H:M) of each record, the ground temperature and position in the vegetation where the insect was collected (Day: ground, canopy, top and flying; Night: ground, canopy, net). Finally, for each insect we recorded the body length, stage (instar three to five and adult), and sex (female, male).

### 2.3 Data analyses

We studied the subhabitat preference of locust in the reed habitat (e.g., ground, canopy, top and flying) in relation to locust traits (development and sex) and time using contingency tables and conditional extended mosaic plots (Zeileis et al., 2007) using the package vcd in R (Meyer et al., 2006). This type of plot shows an area proportional visualization of a table of expected frequencies. It is composed of boxes, with size proportional to the corresponding frequency entry, given the dimensions of previous splits. The shading in the plots visualizes the Pearson residuals representing the standardized deviations of the observed frequencies from expectations. The blue and red colour show respectively significant deviations (*p < 0.05*) above and below the expected frequencies. Finally, we also computed the sum-of-squares test of independence, based on the permutation distribution of 1000 iterations. Significant test (*p < 0.05*) rejects the null hypothesis of independence (Zeileis et al., 2007).

The linear mixed models were used to understand the relation between body temperature and the factors of interest: study area (only day data), development stage (instar-adult), position in vegetation, sex and body length. Prior to the models, we analysed the correlation between the factors to avoid collinearity. Body length was highly correlated with development stages and sex: higher size for females and older stages (see Appendix). Thus, we performed two sets of models, first with sex and stages and second with body length. For the first set of models, differences of internal body temperature during the day across study area (provinces), position, development stage (instar-adult) and sex was compared in a linear mixed model including as random effects site. We also included ground temperature as fixed effect to control for ambient temperature experiencing each individual locust. The distribution of errors was modelled as a Gaussian function with identity link. A similar linear model was implemented for the night survey but only for the study site available, thus without random effect for site. Significant differences among levels of each factor were tested using a post-hoc normal host z test (asymptotic t test) using Tukey procedure. We repeated the same procedure using a similar structure of linear mixed models for day and night datasets but only including body length instead of sex and development stage. All analyses were performed in R 4.2.1 using packages multcomp, lme4 and lmerTest.

## 3 Results

### 3.1 Subhabitat preference of *L. m. manilensis* in natural ecosystem

**FIGURE 2:**
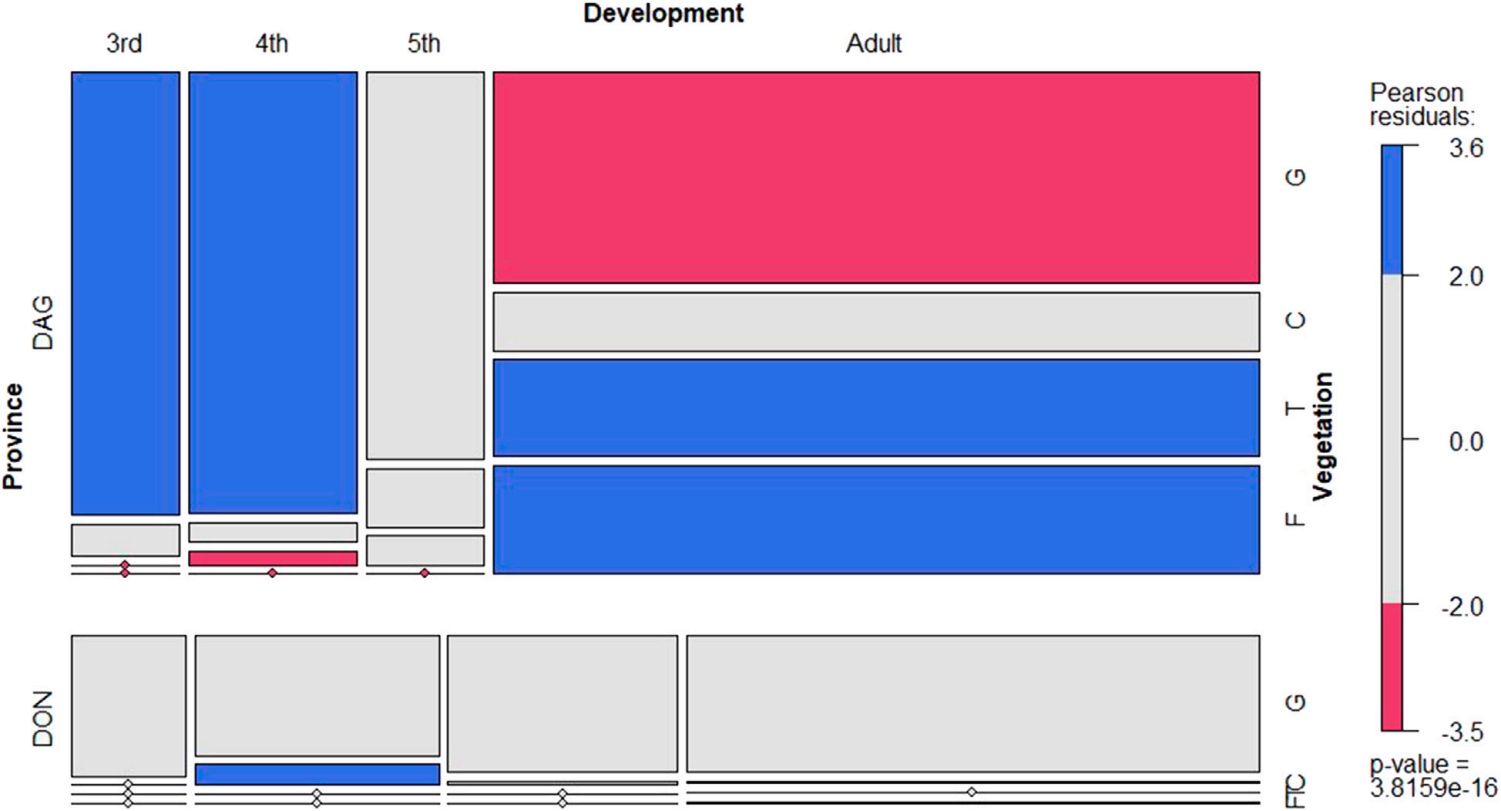
Conditional mosaic plot based on the contingency table with the number of locust records in Dagang (top) and Dongying provinces (bottom) per vegetation position (y-axis) and development stage (x-axis) in daytime. The color gradient indicates the Pearson residuals representing the standardized deviations of the observed frequencies from expectations. The blue and red colors indicate the combination of categories statistically significant at 95% confidence level (α = 0.05) respectively above and below their expected frequencies. Boxes for the vegetation variable are ordered from ground to flying position (G - ground, C- canopy, T-top and F-flying). The *p*-value at the right bottom corner indicates the χ^2^ test of independence, based on the permutation distribution.

*L. m. manilensis* significantly preferred the ground as their major activity subhabitat in both locust breeding regions (Figure 2). Adults showed higher variable preference on their subhabitat significantly preferring higher positions in the vegetation (Figure 2). This pattern was particularly relevant in Dagang while in Dongying most insects preferred the ground irrespectively of their developmental stage (Supplementary Figure S1).

**FIGURE 3:**
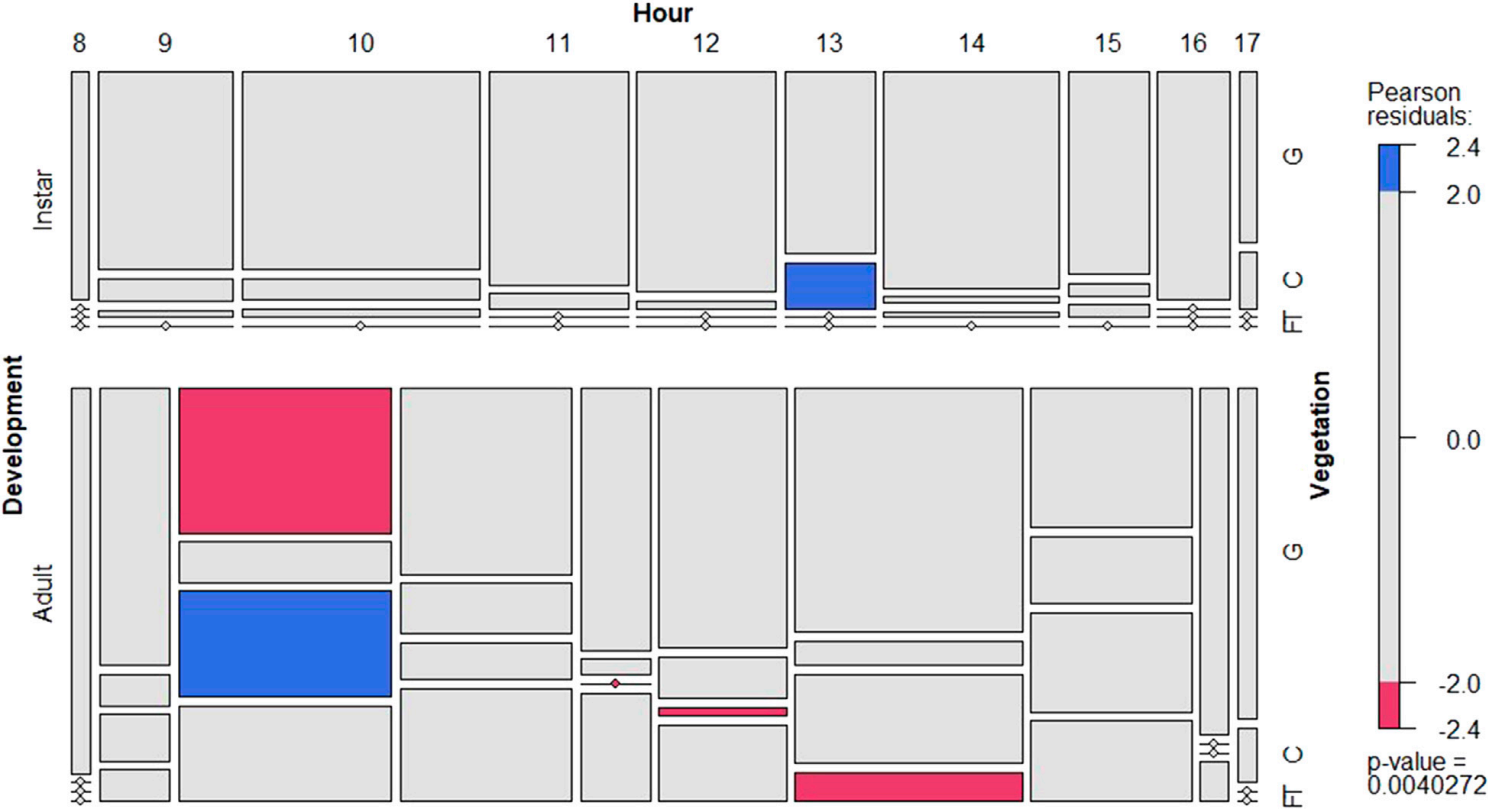
Conditional mosaic plot based on the contingency table with the number of locust records for all instars (left figure) and adults (right) in daytime. For each development group it shows the departure from expectations per vegetation position (y-axis) and hour (x-axis). See details in Figure 2.

The insect behavior was variable during daytime, although there is a significant trend to prefer ground subhabitat particularly for instars (Figure 3; Supplementary Figure S2). Adults showed one clear peak of activity at 10:00 showing significantly less preference for the ground subhabitat. Interestingly, adults showed higher flying activity during midday (Figure 3; Supplementary Figure S2). During night time, the preferred subhabitat for locusts was the plant canopy with a sharp increase towards the ground just before sunrise (Supplementary Figure S3).

**FIGURE 4:**
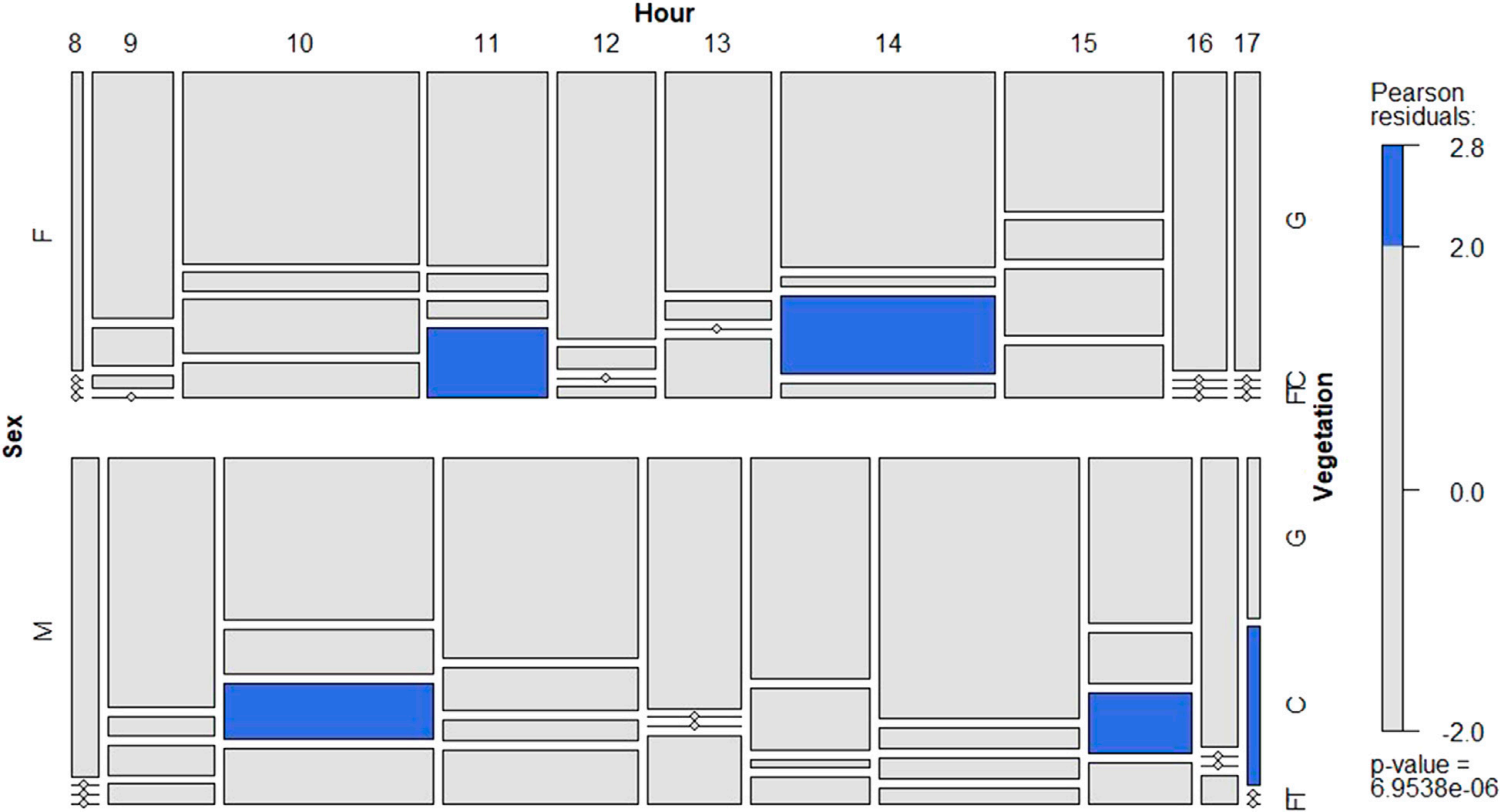
Conditional mosaic plot based on the contingency table with the number of locust records for female (left figure) and male (right) in daytime. For each development group it shows the departure from expectations per vegetation position (x-axis) and hour (y-axis). See details in Figure 2.

During day time, both female and male showed a similar behaviour pattern (Figure 4; Supplementary Figure S3). They tended to significantly prefer higher positions in the vegetation at two peaks (10:00–11:00 h and 14:00–15:00 h). Interestingly, males moved up earlier in the morning and later in the afternoon. During night time, both sexes preferred the canopy as their main subhabitat (Supplementary Figure S4).

### 3.2 The diurnal pattern of internal body temperature of *L. m. manilensis* in two different zones

**FIGURE 5:**
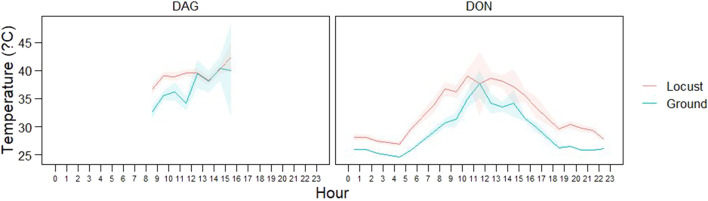
Diurnal pattern of locust body temperature (red) and ground temperature (blue) in the two study areas: Dagang (left) and Dongying (right). For each hour the plot indicates the mean (line) and confidence intervals (i.e., standard error).

The mean body temperature during the day was 38.42°C (±3.52 SD; Min: 28.7; Max: 47.2) while during the night body temperature was 28.23°C (±1.65 SD; Min: 24.7; Max: 37.8). Internal body temperature of *L. m. manilensis* showed a characteristic diurnal pattern reaching rather constant minimum temperatures during the night and reaching maximum peaks at mid-day with high variability (Figure 5). Locust body temperature was consistently higher than ground ambient temperature, particularly during night time (Figure 5). This pattern was consistent in both study areas (Figure 5). According to the linear mixed model, body temperature was significantly related to ground temperature (*p* < 0.0001).

### 3.3 The relationship between locust traits and internal body temperature of *L. m. manilensis*

**FIGURE 6:**
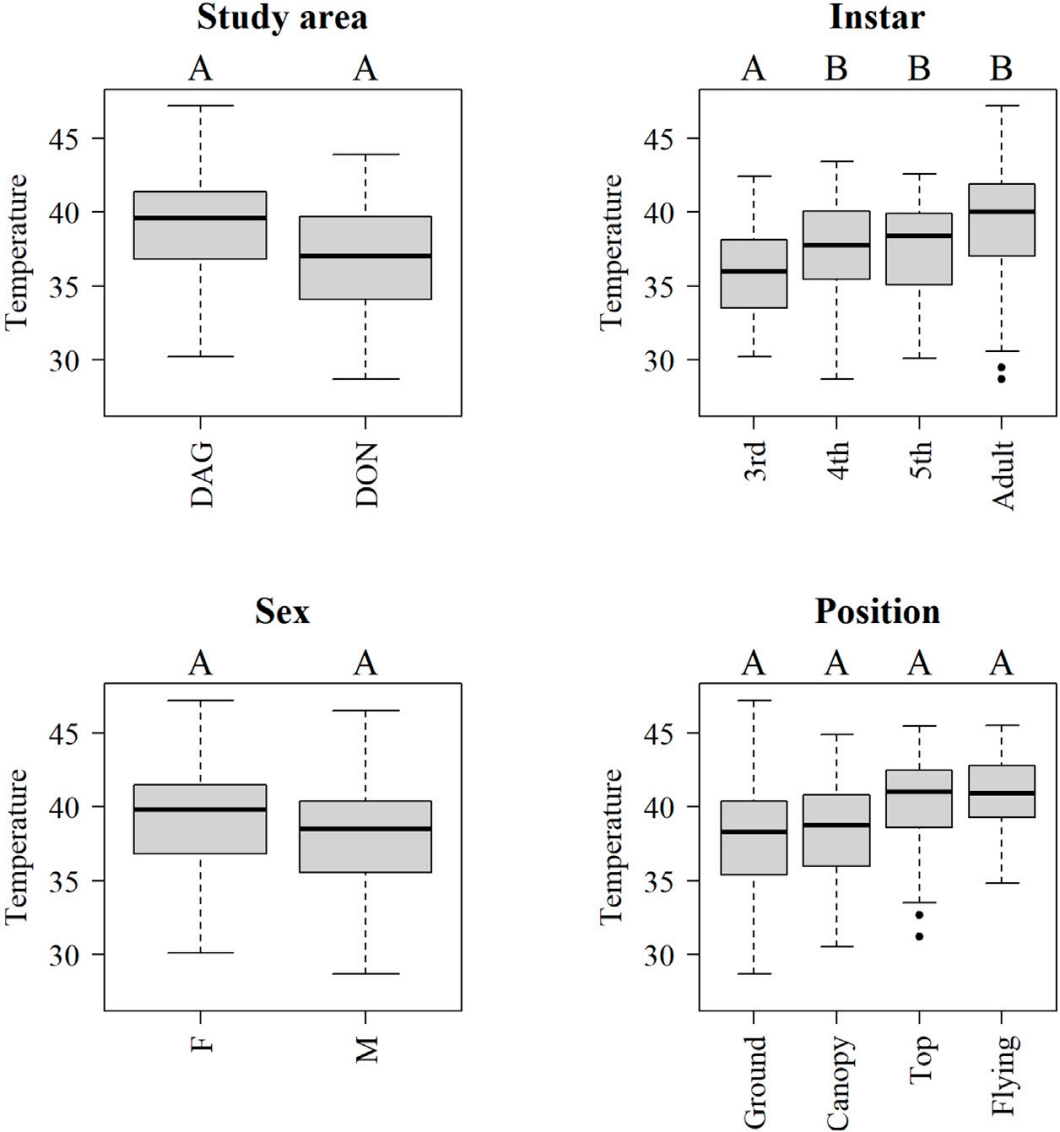
Boxplots of internal body temperature of *Locusta migratoria manilensis* across provinces (DAG-Dagang, DON-Dongying), instar, position and sex (F-female, M-male) at daytime (6:00–18:59). Letters above each level indicate significant differences (*p <* 0.05) between levels in a mixed model including all indicated factors and controlling by ambient temperature and site.

During day time, mean internal body temperature increased with the development stage of the insect (Figure 6). Adults, 4th and 5th instars showed higher temperature than 3rd instars (*p* < 0.05, Figure 6). In contrast, mean body temperature between locust sex, study areas and position at the vegetation did not show significant differences.

**FIGURE 7:**
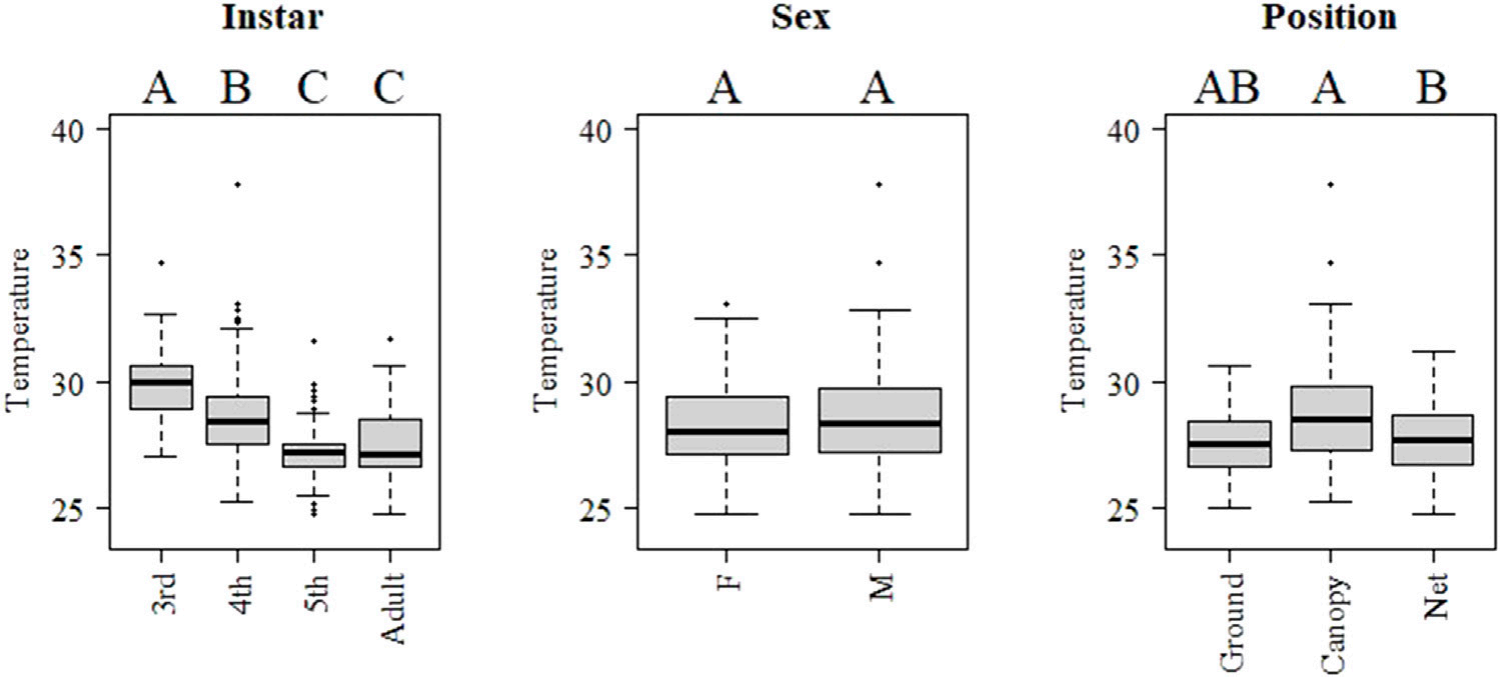
Boxplots of internal body temperature of *Locusta migratoria manilensis*, instar, sex and position at night-time (raw data 19:00–05:59). Letters above each level indicate significant differences (*p <* 0.05) between levels in a mixed model including all indicated factors and controlling by hour.

The pattern during night-time was clearly different than for the day (Figure 7). Body temperature decreased with development stage, with adults and 5th instar showing lower temperature than younger instars. Males showed higher body temperature than females, but there was no significantly difference found. Locusts in the canopy showed higher temperature than at ground (Figure 7). Body and ground temperature were positively related for day (beta: 0.34, t value: 18.57, *p* < 0.001) and night sampling (beta: 0.64, t value: 4.05, *p* < 0.001).

**FIGURE 8:**
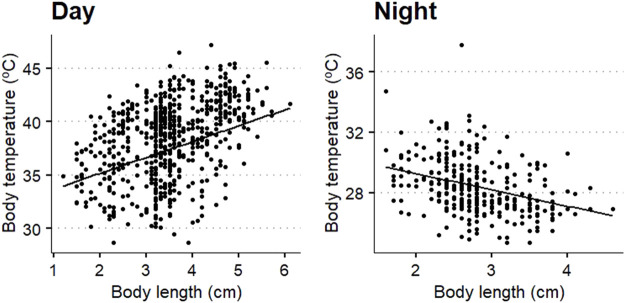
Scatterplots of the relation between body temperature of *Locusta migratoria manilensis* and body length at day (left plot) and night (right plot). Black line shows the fitted linear mixed model for the Dongying region and average ground temperature.

Body length was significantly related to body temperature (Figure 8), with a positive trend during the day (beta: 0.55, t value: 4.07, *p* < 0.001) and negative during the night (beta: −1.08, t value: −6.44, *p* < 0.001).

## 4 Discussion

Ectotherms are intrinsically affected by fluctuations in ambient temperature, as the latter determines the rate of biochemical and physiological reactions including the development of both the host and the pathogen. Thus, the study of body temperature in insects is not only important for ontogeny, but also critical for improvement in the control effect of biopesticide and sustainable pest management. Previously studies implied that the body temperature of *L. m. manilensis* was a similar tendency for variation of natural subhabitat ground temperature (Liu et al., 2018). Similarly, the body temperature of *Oedaleus decorus asiaticus* (Bey-Bienko) increased/decreased with increases/decreases inground temperature, respectively during the daytime (Cheng et al., 2022). This study identified under natural conditions, how subhabitat and species traits modulate *L. m. manilensis* internal body temperature across two separate study breeding regions in China. These results are extremely relevant to improve tailored biopesticide applications taking advantage of habitats frequented and traits that modulate internal body temperature.

The results showed that *L. m. manilensis* preferred the ground as their major activity habitat regardless of the locust breeding regions. Only adults showed a distinct pattern to move upwards in the vegetation during short periods of time during the day. This behaviour is essential to survive as they have limited capacity to internally regulate their body temperature and mainly rely on the surrounding environment (Angilletta, 2009). During the day the Oriental migratory locusts showed two peaks of activity where it tended to climb the vegetation. Specifically, locust chose to move up after sunrise resting at mid and top of the canopy, and then moved back to the shade at noon possibly to avoid injury due to high temperature. Finally, once the ambient temperature was cooling down, the locusts left the ground and moved up in the canopy with a peak around 15:00 h in the afternoon. This behavioural pattern is characteristic of other diurnal insects. For instance, Ellis and Ashall, (1957) noted that bands of hoppers of *S. gregaria* had a definite diurnal pattern of behavior. Before dawn, the hoppers were mainly roosting and again during the hottest part of the day, most of them roosted (Ellis and Ashall, 1957). Chapman (1959) also found a similar pattern for Red locust, *Nomadacris septemfasciata* (Serville)*.* In the morning and evening, all the locusts inhabit higher plants, which may be related to the increase of light intensity and the decrease of environmental temperature.

During the night, locusts in this study preferred the canopy as main resting habitat. In a similar context to our study, Cheng et al. (2020) found that the position of the Oriental migratory locust was different in different times of the day. In contrast to daytime, locusts at night preferred the canopy as resting subhabitat. Specifically, Cheng et al. (2020) found that during night, the Oriental migratory locust preferred to choose the place higher than 60 cm. Chapman (1959) also found that *N. septemfasciata* inhabited higher plants all night. Similarly, the hoppers of Moroccan locust, *Dociostaurus maroccanus* (Thnb.), remained on or near the ground during the day, moving to the top of the plants at night (Uvarov et al., 1951). In contrast, Senegalese grasshopper, *O. senegalensis* (Krauss), was observed feeding in the evening, and spent the night on the ground in India desert (Chandra, 1983).

Regarding sex differences in habitat preference, this study results implied that both female and male tended to show similar behaviour. However, the proportion of female adults preferred to the lower part of the plant comparing the male adults of *O. d. asiaticus* and *Calliptamus abbreviates* (Ikovnnikov). The female adults of *Euchorthippus unicolor* (Ikonnikov) preferred to choose the upper and top parts of plants more than male adults (Bai et al., 2014). The reason would be related to the different locusts requesting different habitat and hosts. The physiological needs driven their movement. In general, the locusts were plentiful in breeding grounds in the plain where female locusts were ovipositing (Uvarov et al., 1951).

The differences in internal body temperature are mainly related to the species traits (i.e., size and development stage) irrespectively of the breeding region or position preference at reed vegetation. Both selected breeding regions, North Dagang Reservoir area at the southeast of Tianjin City and the Yellow River Delta area, are two of the main areas in east coast China prone to the migratory locust plagues, where the central government and local governments established long-term monitoring and management scheme. Despite the distance between the regions, both areas share similar vegetation, geographical context and availability of natural breeding habitat determining the occurrence of the oriental migratory locust. Thus, the physical process that links ambient and internal body temperature through locust morphological traits seems to be constant across these characteristic locust breeding regions in east coast China. This pattern implied that the comprehensive body temperature of locust in one habitat region may represent the cases in similar habitats, and can provide enough fundamental information to establish the biopesticide model to precisely control.

The main factor affecting the average body temperature of locusts was development stage. At night, position at the vegetation was also a key factor affecting the average body temperature of locusts. These patterns arose once fluctuation in ambient temperature was controlled (i.e., ground temperature) indicating that endogenous traits such as development age have a clear relevance in how locust thermoregulates in relation to the surrounding temperature. Thomas and Blanford. (2003) also identified that some internal characteristics of insects can affect insect thermoregulation, such as body length, age, sex, colour. Similarly, we also identified a significant relation between body length of *L. m. manilensis* and internal body temperature. In fact, body length or size are factors to absorb the energy from the ambient. Body size is a major trait that impacted on nearly all aspects of an individual’s life history (Woodward et al., 2005; Brose, 2006). Furthermore, as ectotherms increase in size, solar radiation will raise their body temperature further above the ambient temperature due to the reduced effect of convection on larger organisms (Porter and Gates, 1969; Stevenson, 1985). This explanation is plausible for the pattern observed during daytime, where bigger individuals heated by the Sun light showed higher average body temperature than smaller ones. In adults, the prediction of increased body temperature with increased size has been confirmed by comparison across many different insect species (Willmer and Unwin, 1981). As for developmental size change, early work on the desert locust, *S. gregaria*, suggested increased body temperature between the first and last instars (Stower and Griffiths, 1966), and more recent work on *Manduca sexta* (L.) clearly demonstrates this pattern across all larval stages (Woods, 2013).

We would expect a similar pattern between body temperature and size during the night time. As body size affects the rate of heating and cooling, larger animals should show higher thermal inertia than smaller ones (Stevenson, 1985). Thus, we would expect larger animals that have been heated during the day to keep their temperature higher than smaller individuals. Nevertheless, the night-time data showed the opposite pattern, with a decrease in body temperature with body size. A possible explanation for this finding is the behavioural change across development stages. In fact, not only does body size directly affect body temperature (Stevenson, 1985; Woods, 2013), but size should also alter thermoregulatory behaviour, with subsequent indirect changes in body temperature. It is possible that adults, with higher mobility than other instars, are able to track changes in microhabitat temperature moving to specific areas where temperature is more suitable for development or indirectly to escape predators.

The activity pattern may provide the useful information for field spray good timing. We have identified main characteristics in daily variation of *L. m. manilensis* subhabitat preference and the main factors affecting their internal body temperature. Based on these findings, the application of biopesticides should focus on younger locusts at dawn or dusk when temperatures tend to be lower and temperatures more suitable for EFP development. This will help biopesticides play their roles and minimize the risk of outbreak escalation. In addition, the advanced technology against crop insect pests and diseases including the Earth observation, agricultural Apps, generally demanded the ground truth data of the pests. It is not possible to carry out all the collation everywhere, thus the results of this study contribute to the efficacy of biopesticides, and predict such variability in the performance of fungal biopesticides increasing confidence in their use.

## Data Availability

The raw data supporting the conclusions of this article will be made available by the authors, without undue reservation.
