# Supplementary material for: Daily activity patterns and body temperature of the Oriental migratory locust, Locusta migratoria manilensis (Meyen), in natural habitat
Source: Front Physiol. 2023 Feb 3;14:1110998. doi: 10.3389/fphys.2023.1110998 (PMC9936075; doi:10.3389/fphys.2023.1110998)
Supplement: Supplementary file 1 [file DataSheet1.pdf]

Supplementary Material

Daily activity patterns and body temperature of the oriental migratory locust, *Locusta migratoria manilensis*, under natural habitat

Hongmei Li[1,2*], Jingquan Zhu[3], Yumeng Cheng[1], Fuyan Zhuo[3], Yinmin Liu[1], Jingfeng Huang[4], Bryony Taylor[5], Belinda Luke[5], Meizhi Wang[1], Pablo González-Moreno[5,6]

*** Correspondence:** Corresponding Author: h.li@cabi.org

APPENDIX

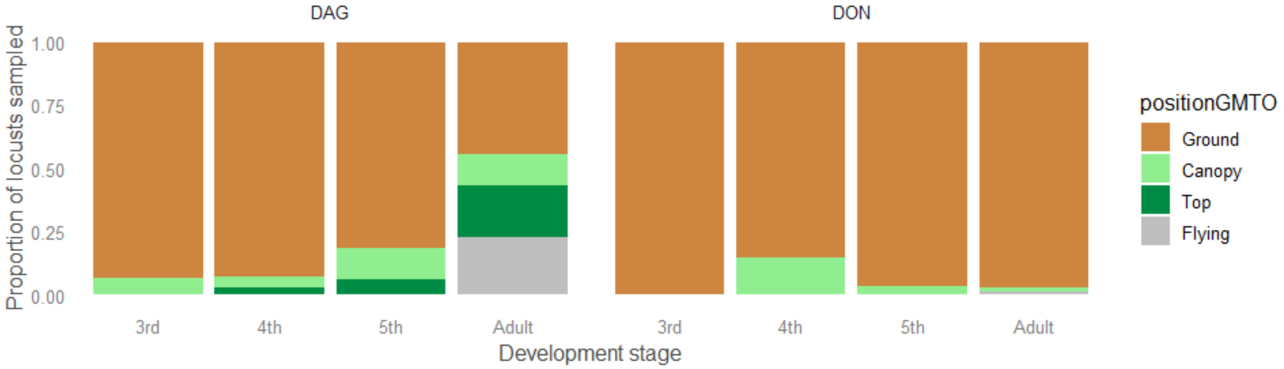

Supplementary Figure 1 Proportion of locust sampled during day time at each vegetation position (Ground, canopy, top and flying) per province (DAG-Dagang, DON-Dongying) and development stage

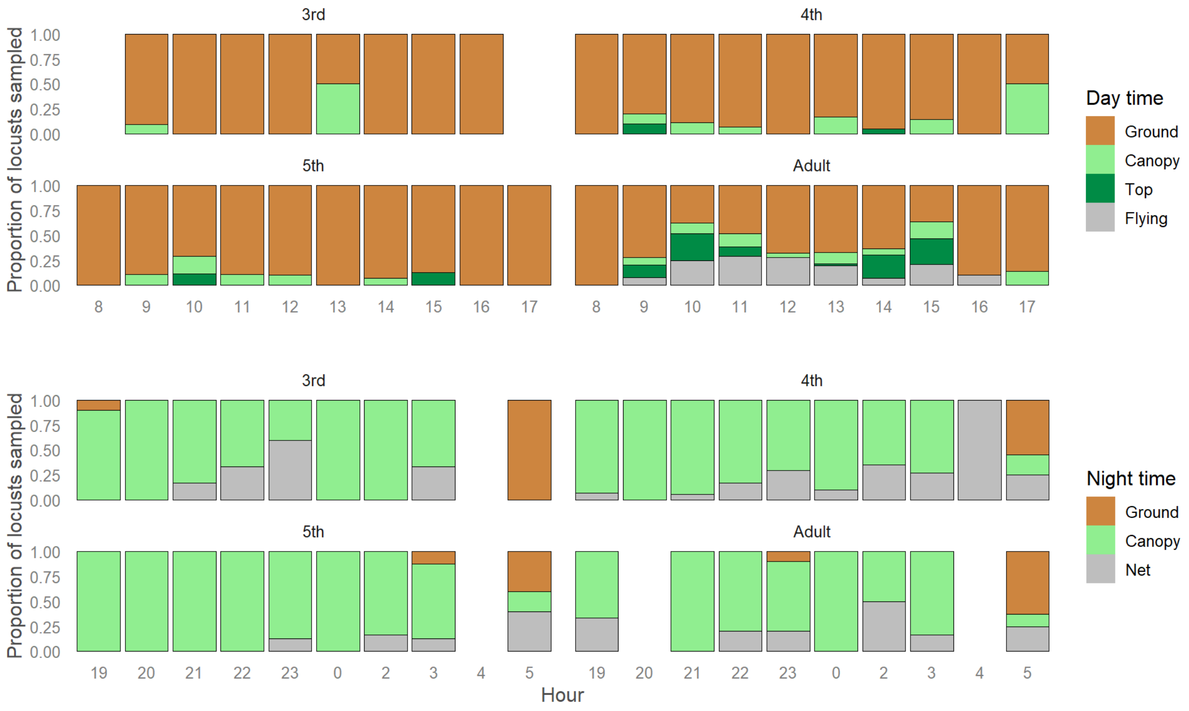

Supplementary Figure 2 Proportion of locust sampled in each vegetation position (Ground, canopy, top and flying) per hour and development stage for day time in open field conditions (top) and night time in outdoor cage (bottom)

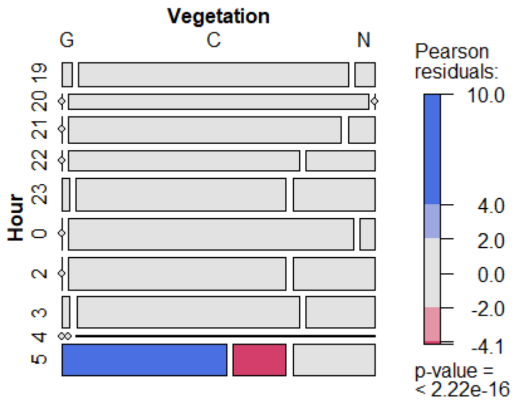

Supplementary Figure 3. Mosaic plot based on the contingency table with the number of locust records per vegetation position and hour during night time. The color gradient indicates the Pearson residuals representing the standardized deviations of the observed frequencies from expectations. The blue and red colors indicate the combination of categories statistically significant at 95% confidence level ($\alpha = 0.05$) respectively above and below their expected frequencies. Boxes for the vegetation variable are ordered from ground to flying position (G - ground, C- canopy, T-top and F-flying). The p-value at the right bottom corner indicates the χ^2 test of independence, based on the permutation distribution

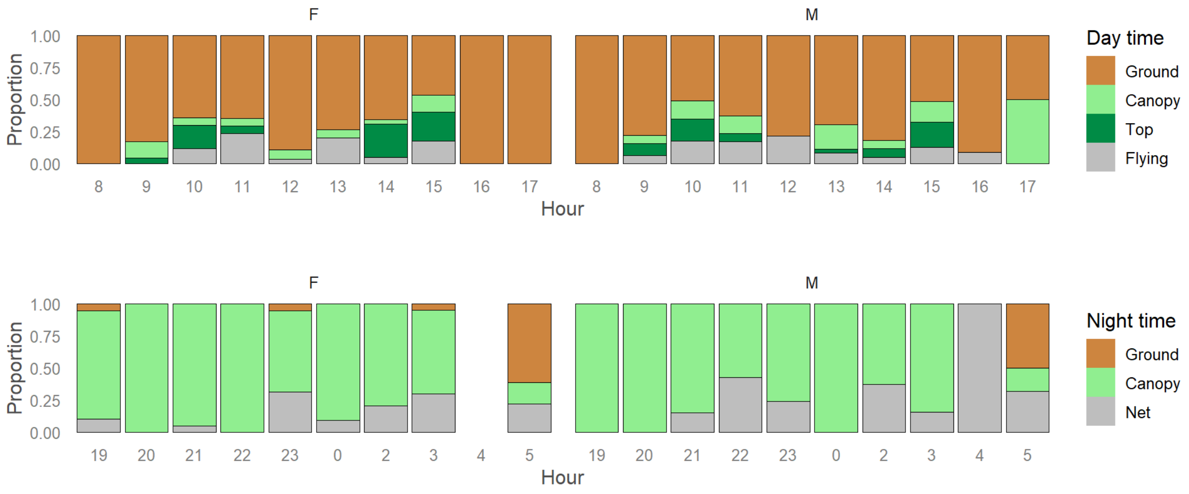

Supplementary Figure 4 Proportion of locust sampled in each vegetation position (Ground, canopy, top and flying) per hour and sex (F- female, M-male) during for day time in open field conditions (top) and night time in outdoor cage (bottom)
